# FusionSense: Emotion Classification Using Feature Fusion of Multimodal Data and Deep Learning in a Brain-Inspired Spiking Neural Network

**DOI:** 10.3390/s20185328

**Published:** 2020-09-17

**Authors:** Clarence Tan, Gerardo Ceballos, Nikola Kasabov, Narayan Puthanmadam Subramaniyam

**Affiliations:** 1 Knowledge Engineering and Discovery Research Institute, Auckland University of Technology, Auckland 1010, New Zealand; nkasabov@aut.ac.nz; 2 School of Electrical Engineering, University of Los Andes, Merida 5101, Venezuela; gerardoacv@gmail.com; 3 Faculty of Medicine and Health Technology and BioMediTech Institute, Tampere University, 33520 Tampere, Finland; narayan.subramaniyam@tuni.fi; 4 Department of Neuroscience and Biomedical Engineering, School of Science, Aalto University, 02150 Espoo, Finland

**Keywords:** facial emotion recognition, Evolving Spiking Neural Networks (eSNNs), Spatio-temporal data, NeuCube, multimodal data

## Abstract

Using multimodal signals to solve the problem of emotion recognition is one of the emerging trends in affective computing. Several studies have utilized state of the art deep learning methods and combined physiological signals, such as the electrocardiogram (EEG), electroencephalogram (ECG), skin temperature, along with facial expressions, voice, posture to name a few, in order to classify emotions. Spiking neural networks (SNNs) represent the third generation of neural networks and employ biologically plausible models of neurons. SNNs have been shown to handle Spatio-temporal data, which is essentially the nature of the data encountered in emotion recognition problem, in an efficient manner. In this work, for the first time, we propose the application of SNNs in order to solve the emotion recognition problem with the multimodal dataset. Specifically, we use the NeuCube framework, which employs an evolving SNN architecture to classify emotional valence and evaluate the performance of our approach on the MAHNOB-HCI dataset. The multimodal data used in our work consists of facial expressions along with physiological signals such as ECG, skin temperature, skin conductance, respiration signal, mouth length, and pupil size. We perform classification under the Leave-One-Subject-Out (LOSO) cross-validation mode. Our results show that the proposed approach achieves an accuracy of 73.15% for classifying binary valence when applying feature-level fusion, which is comparable to other deep learning methods. We achieve this accuracy even without using EEG, which other deep learning methods have relied on to achieve this level of accuracy. In conclusion, we have demonstrated that the SNN can be successfully used for solving the emotion recognition problem with multimodal data and also provide directions for future research utilizing SNN for Affective computing. In addition to the good accuracy, the SNN recognition system is requires incrementally trainable on new data in an adaptive way. It only one pass training, which makes it suitable for practical and on-line applications. These features are not manifested in other methods for this problem.

## 1. Introduction

The central aim of affective computing is to enable seamless communication between humans and computers by developing systems that can detect and respond to the various affect states of the humans [1]. Affective computing is an interdisciplinary field of research that involves experts from computer science, psychology, social, and cognitive sciences. Affect recognition has important applications in several fields, such as medicine [2], driver fatigue monitoring, human-computer interaction, sociable robotics [3], and security systems, to name a few.

Modelling affect can be classified into three categories: categorical, dimensional, and components. Categorical models classify emotions into a set of discrete classes, which are easy to describe and these include six basic emotions, such as happiness, sadness, fear, anger, disgust, and surprise. Owing to its simplicity, categorical models have been extensively utilized in affect research. In contrast, dimensional models represent emotion as a point in multidimensional space, where the dimensions include valence, activation, and control, allowing for the description of more complex and subtle emotions. However, such multidimensional space can pose a significant challenge to automatic emotion recognition system and, thus, researchers have mostly used the simplified two-dimensional model of arousal and valence proposed in [4], where arousal ranges the intensity of emotion from calm to excited, and valence ranges from unpleasant to pleasant [5]. Finally, the component model of emotions arrange emotions in a hierarchical fashion, where complex emotions can be derived from the combination of a pair of basic emotions. The most popular component model proposed by Plutchik [6] is based on evolutionary principles and it has eight basic bipolar emotions.

Affect can be expressed via facial expression, body movements, voice behavior, gestures, and an array of physiological signals, such as heart rate, sweat, pupil diameter, brain signals, to mention a few. The problem of recognizing emotions by utilizing facial expressions from videos and static images have been addressed by several studies [7,8,9]. Advances in deep learning methodologies have created huge interest in application of such methods in facial emotion recognition (FER) [10,11,12,13,14], most of which are based on supervised learning. The methods do not allow for incremental, adaptive learning on new data, and they are not suitable for on-line applications. For an excellent overview of the application of deep learning and as well as shallow learning approaches to FER, the reader is directed to [15] and the references there in. Additionally, the reader can refer to this Chapter on Multimodal Affect Recognition in the Context of Human-Computer Interaction [16].

Spiking neural networks (SNNs) represent the third-generation of neural networks, modelling neurons and interactions between them in a biologically more realistic manner as compared to second-generation neural networks based on ANNs. SNNs are an ideal choice to handle the emotion recognition task from video data, given their ability to effectively handle spatio-temporal data [17] (see Section 4 for details).

In this work, we propose building an emotion recognition system for multimodal data. system using SNNs. To this end, we use the NeuCube framework [18], which is a type of evolving SNN (eSNN). In this paper, we develop an encoding method to map the continuous facial feature values to spikes based on population coding. We use the data from Mahnob-HCI dataset to test the NeuCube framework for the classification of binary valence in response to video stimuli.

The structure of the paper is organized, as follows. In Section 2, we provide some background literature on various data modalities used in affect detection, where, as in Section 3, we describe strategies for multimodal data fusion. In Section 4 we provide some background on SNN and the NeuCube framework. Section 5 details the methodology used in our work and Section 6 presents the results. In Section 7, we discuss our results and, in Section 8, the direction for future work is presented and it concludes the paper.

## 2. Signals for Affect Detection

### 2.1. Facial Expression

One of the immediate and natural ways for humans to communicate their emotions is through facial expressions, which constitute about 55% of the information communicated during face to face human interaction [19]. Thus, affect research has primarily focused on detecting emotions from the face. Research on facial emotions have shown that the six basic emotions, such as fear, anger, sadness, enjoyment and disgust can be detected with facial expressions [20,21] and dectecting an emotion is equivalent to detetecting the associated prototypic facial expression. Based on the Facial Action Coding System (FACS), which originally described 44 single action units (AU) including head and eye movements, with each action unit linked with an independent motion on the face and the correponding muscles, for example lip suck motion with the muscle orbicularis oris [22]. Several deep learning techniques have been used to build automatic facial emotion recognition (FER) system, including deep boltzmann machine (DBM), deep belief networks (DBNs) [23,24,25], convolutional neural networks (CNNs) [11,26,27,28,29], auto-encoders [30,31,32], and recurrent neural networks (RNNs), to mention a few.

### 2.2. Speech

Affective information from speech can contain lingusistic and paralinguistic features, which refer to what is said and how it is said, respectively. Although speech is a fast and efficient method of communication that can be exploited in affect research, detecting the emotional state of the speaker using speech signal is still a significant challenge. There is no clarity on which features of the speech signal are most powerful is distinguishing different emotions. It has also been shown that, as compared to facial expressions, the accuracy of affect detection from speech is lower [33]. For instance, the basic emotions, such as sadness, anger, and fear, can be recognized using speech, where as disgust is hard to detect [1]. Moreover, cultural differences among speakers has not been addressed thoroughly with most of the affect research involving speech focusing on monolingual emotion classification [33]. The features that are typically extracted from speech signal include both global and local features, Local features refer to pitch and energy extracted from small segments, into which a speech signal is typically divided to make it stationary, whereas global features refer to statistics of all the local features extracted from a long signal. Studies have shown that global features have better classification accuracy than local features [34,35,36]. However, studies have shown that global features cannot distinguish between emotions that have similar arousal [37] and may prove to be sub-optimal when using classifiers, such as Hidden Markov Model (HMM) and Support Vector Machines (SVM), due to insufficient number of training vectors [33]. Because the properties of the different speech sounds can be altered by different emotions, some studies have also explored the benefits of phoneme-level modeling for the classification of emotional states from speech rather than using the prosodic features, such as pitch and energy [38]. Their results showed that the using phoneme-class classifiers outperformed HMM classifiers just based on global features. Apart from using HMM or SVM classifiers, several deep learning techniques have been explored for emotion recognition from speech signals including DBM [39,40], auto-encoders [41,42], DBNs [43,44], and CNNs [45,46,47], to cite a few. Despite the aforementioned challenges, speech is still an important signal that can be used for affect detection, as it is non-intruive and has high temporal resolution.

### 2.3. Posture and Body Movements

In comparison to speech and facial expression, perceiving emotions through body movements and postures is a relatively less explored topic in affect research. In fact, 95% of the literature in research on human emotions focuses on facial expressions and less than 5% on speech and other physiological signals with the remaining little of body movements. Several studies in the past have shown that body movements and postures can contribute to the recognition of emotional states [48,49], with perhaps the most influential work in this topic dating back to the second half of 19th century by Charles Darwin [50]. Body postures may offer certain advantages in affect detection given the multiple degrees of freedom human body possesses, which can aid in communication of emotions and subsequently affect detection, even at long distances, at which facial emotions are unreliable [51], indicating that postures contain information not present in facial expressions. Another advantage of posture-based affect system could be that, in comparison to facial expression, which may be intentionally controlled, postures and body movements are unintentional and, thus, less susceptible to social editing [1]. In a study on deception by Eckman and Friesen [52], it was shown that liars were less successful at deception through body movements as compared to more controlled channels of communication, such as facial emotions, which they referred to as nonverbal leakage. Gestures, which can be defined as collection of body movements or actions involving head, hands, and other parts of the body allow the communication of a range of thoughts and emotions. Some of the basic gestures have been shown to be similar across the cultures. Given the advantages of this non-verbal communication channel, relatively few studies have utilized deep or machine learning framework to recognize emotions using body movements, postures, and gestures [53,54].

### 2.4. Physiological Signals

Physiological signals such as electroencephalography (EEG), electrocardiogram (ECG), electromyogram (EMG), skin conductance, also known as Galvanic skin response (GSR), skin temperature, as well as pupilary diameter can be used for affect detection, apart from the above mentioned non-physiological signals. Physiological signals for affect detection are typically acquired in a non-invasive manner using wearable sensors. Heart rate (HR) and hear rate variability (HRV) can be derived from ECG signals. Skin temperatue has been shown to be a effective indicator of the emotional state as shown in [55] and it primarily reflects the activity of the autonomic nervous system (ANS). Another modality that captures the activity of ANS is the GSR or skin conductance, which can be obtained by measuring the electrical potential on the skin after passing a negligible amont of current. GSR is considered to be a reliable indicator of arousal [56], as it captures the activity of the sweat glands on the skin.

In affect research, ECG signals are typically recorded by a pair of electrodes, which are a subset of lead I configuration comprising of 12 electrodes. Features such as HR and HRV can be further derived from ECG that can reflect the activity of the sympathetic and parasympathetic branch of ANS system. HR and HRV have both been used in several studies to asses the mental states of the subject [57,58]. An EMG signal is reflective of the strength of muscle movements and is typically recorded by a pair of electrodes placed on the body. Studies have shown that when the subject is under some emotional stress, the changes in the facial expression can be measured using EMG activity [59,60]. Apart from using electrodes on the face, other studies have also looked into measuring the activity of jaws or shoulders in order to identify emotional states [61].

Breathing is another physiological process that is shown to be altered by basic emotions, such as happiness, sadness, and anxiety [62]. Researchers have observed rapid breathing during arousal state [63] and as well as changes in respiratory pattern of subjects looking at photographs that induce emotions [62]. The respiratory rate is shown to be modulated by emotions, particularly anxiety affecting the expiration rate [62], where timing and volumetric aspects of breathing are altered by various physical and mental stress [64].

Finally, EEG is probably the most widely used physiological signal to study emotion. EEG is a low cost technology a compared to other neuroimaging modalities and has very good temporal resolution. EEG electrodes record the activity of a large number of synchronous neurons as potential difference on the scalp. Several studies have utilized EEG for emotion recognition [65,66,67] and classification of emotional states of arousal, valence and dominance. In addition to EEG, pupilary diameter size is also an indication of emotional state, with several studies reporting that the size of the pupil discriminates during and after different kinds of emotional stimuli [68,69].

Several deep learning methodologies have been utilized for emotion recognition using physiological signals [70,71,72,73]. The reader is directed to [1] for an exhaustive list of literature.

## 3. Multimodal Affect Recognition

Although a majority of the machine learning and deep learning framework for affect recognition uses data from one modality, i.e., video or audio or EEG, recently there has been considerable interest in fusing data from the above mentioned modalities. Multi-sensor data fusion can be highly advantageous in terms of improving the reliability and accuracy of affect detection and, furthermore, multimodal systems have shown to outperform unimodal system as discussed in [74]. Multimodal fusion involves combining data from many different types of sensors and such fusion can be primarily performed at two distinct levels, known as feature-level fusion and decision-level fusion.

### 3.1. Feature-Level Fusion

In the feature-level fusion approach (also known as early fusion), features that are derived from different modalities are combined into a single feature vector, on which a classifier can then be trained. It is well known that humans use and integrate multiple sensory cues during face-to-face interaction to detect affective states and is the fundamental idea behind feature-level fusion [75]. The main advantage of feature-level fusion is that correlation between multimodal features at an early stage can lead to better performance, requiring only one learning phase on the feature vector. Several studies have utilized this approach for affect research [8,76,77]. However, feature-level fusion also has several challenges. Because features obtained from different modalities can have different time-scales, achieving time synchronization to bring the features in same format can be difficult and computationally expensive. Additionally, given the large feature set that one obtains with feature-level fusion, the classification accuracy can be severly affected if the training dataset is limited. Furthermore, learning cross-correlation between the heterogenous features can prove to be difficult [78].

### 3.2. Decision-Level Fusion

In the decision-level fusion approach (also known as late fusion), first the decisions based on features derived from each modality is obtained separately. A fused decision vector is then obtained using the local decisions, which can be used to obtain the final decision or classification [78]. The fundamental advantage of decision-level fusion over feature-level fusion is that the decisions all have the same format and, hence, can be fused easily, thus avoiding synchronization issues. Furthermore, using decision-level fusion allows for the application of optimal classifier or method suited for each modality, thus providing more flexibility when compared to feature-level fusion [79]. Several studies have utilized decision-level fusion for affect research [80,81,82] and it has been noted that researchers prefer decision-level fusion over feature-level fusion [79].

## 4. Spiking Neural Networks

Human brains encode information via discrete events that are known as action potentials or spikes, following an all-or-none principle, where a neuron fires an action potential if the stimulus crosses a certain threshold, else it remains silent. Due to this binary nature of information representation, the human brain still outperforms the existing artificial neural networks (ANNs) in terms of both energy and efficiency [83,84]. When compared to the traditional ANNs, spiking neural networks (SNNs) utilize a more biologically realistic model of neurons [85], thus further bridging the gap between neuroscience and learning algorithms. SNNs have shown the ability to integrate information from different dimensions, such as time, phase, frequency, as well as handle large volumes of data in an adaptive and self-organized manner [17,86], making them particularly suitable to solve online spatio-temporal pattern recognition. SNNs have been shown to be computationally more efficient than ANNs both theoretically [87,88] and in several real-world applications [89]. SNNs have been used in several real-world learning tasks such as unsupervised classification of non-globular clusters [90], image segmentation and edge detection [91], epileptic seizure detection with EEG [92]. Furthermore, Bohte and colleagues devised a supervised learning rule for the SNNs and demonstrated its application in the XOR classification problem and several other benchmark datasets [89]. The evolving SNN (eSNN) is a class of SNN that utilizes rank order learning [93] and was first proposed in [94]. The eSNN handles spatio-temporal data by increasing the number of spiking neurons in time to learn temporal patterns from data [95]. In addition to the open evolving structure of eSNNs that facilitates the addition of new variables and neuronal connections, eSNN have the advantage of fast learning from large amounts of data and they can interact with other systems actively. eSNNs also allows for the integration of various learning rules, such as supervised learning, unsupervised learning, fuzzy rule insertion, and extraction, to mention a few and they are self-evaluating in terms of system performance. These aforementioned properties constitute the evolving connectionist systems (ECOS) principles on which the eSNN is based [96].

Because, in the rank-order learning scheme, the synaptic weights are adjusted only once making it not very efficient for spatio-temporal data, where there may be a need to adjust synaptic weights that are based on the spikes arriving on the same synapse over time. To overcome this disadvantage, an extension of eSNN, known as dynamic eSNN (deSNN), was introduced in [97], which combines rank-order learning with temporal learning rules, such as spike-timing dependent plasticity (STDP), which allows for dynamic adjustment the synaptic weights. However, both eSNN and deSNN do not encapsulate the structural information of the brain in terms of neuronal locations and their connectivity, which may be crucial for modeling spatio-temporal data. The NeuCube architecture, first proposed in [98], aims at building a eSNN that incorporates structural as well as functional aspects of the brain along with utilizing STDP learning rules. The following section gives a brief introduction to the NeuCube architecture. The reader is directed to [18,98,99] for a more detailed introduction.

### 4.1. NeuCube

It is well known that the information in human brain is processed at different spatiotemporal levels, ranging from molecular information processing to higher order cogitive processes. The data can be acquired at different levels of these spatiotemporal processes and an efficient learning method should be able to handle complex spatio-temporal relationship from brain data at different levels. Some examples of spatio-temporal brain data (STBD) include EEG, functional magnetric resonance imaging (fMRI), diffusion tensor imaging (DTI), and positron emission tomography (PET) to mention a few. Traditional methods such as support vector machines (SVM) or multilayer perceptron neural networks (MLP) typically deal with the spatial or temporal aspects of brain data and cannot handle the dynamic interaction between these processes [99]. Furthermore, they cannot incorporate any structural prior knowledge of the brain or handle multimodal brain data. NeuCube [18,98,100] and aslo [18,96,100,101] is a variant of eSNN, initially proposed to handle problems of spatio-temporal pattern recognition in brain data such as EEG, functional magnetic resonance imaging (fMRI) to cite a few, has been further developed to handle various other types of spatio-temporal data, such as audio-visual data, climate data, seismic data, and ecological data [101]. The typical framework of the NeuCube system comprises of

1. An input encoding module, which converts the STBD into trains of spikes that captures temporal patterns present in the data. Various methods have been proposed to achieve this, including population coding [102], address event representation [103], and Bens Spike algorithm [97].
2. A three-dimensional SNN reservoir (3D-SNNr), which takes the spike trains as input. The 3D-SNNr contains neurons that have pre-defined spatial co-ordinates and are modelled as leaky integrate and fire neurons. The initial structural connections between the neurons can be established in several ways, including small-world organization [104] or based on the DTI data. Several studies utilizing EEG, fMRI, and MEG have demonstrated the presence of small-world connectivity in the brain [105,106] and, thus, this is the preferred initial setup for the spatial structure of 3D-SNNr. Based on the temporal association between the input spikes, connections between the neurons is modified while using the spike timing dependent plasticity (STDP) rule. This is a deep unsupervised learning, as deep connectionist structures of many neurons are created as a results of the learning in space and time [96].
3. A classification module, which takes the spiking patterns from 3D-SNNr as its input to perform classification.
4. An optional, Gene Regulatory Network (GRN) for controlling the learning parameter and optimization of 3D-SNNr, exploiting the fact that spiking activity is influenced by the gene and protein dynamics.

The details on the implementation of NeuCube for this study is further described in Section 5.6.

## 5. Methods

### 5.1. Mahnob Database

The MAHNOB-HCI dataset is a multi-modal database for affect recognition and implicit tagging [107]. In this database, 27 subjects (16 females and 11 males) aged between 19 and 40 years old were monitored while watching 20 stimulus clips (34.9 to 117 s long) that were extracted from Hollywood movies and video websites, such as www.youtube.com and blip.tv. The face video, audio and elicited physiological signals (EEG, ECG, respiration amplitude, skin temperature, GSR, and gaze data) were acquired while watching the clips. The ECG signal was obtained by subtracting a measurement from the upper left corner of chest, under the clavicle bone, from that one on left side of abdomen, below the last rib. The respiration signal was obtained by a belt placed in the subject’s abdomen, skin temperature was acquired by a temperature sensor placed at the subject’s little finger and GSR was obtained by passing a negligible current between the electrodes on the distal phalanges of the middle and index fingers of the subject. Gaze data were acquired with Tobii X1205 eye gaze tracker providing position of the projected eye gaze on the screen (at 60 Hz), the pupil diameter, the moments when the eyes were closed, and the instantaneous distance of the subject’s eyes to the gaze tracker device.

Physiological signals, except the gaze data, were acquired at a sampling rate of 1024 Hz (down sampled to 256 Hz for further analysis), while six different views of subject’s facial expressions were simultaneously recorded by six video cameras at 60 fps. In this work, the video taken only by the color camera above the screen were used. After watching each stimulus, the participants used a keyboard interface for answering five questions that were related to emotional label, arousal, valence, dominance, and predictability. The participants answered each question using nine numerical keys, selecting nine emotional labels for the first question and nine possible levels for the last question. In this work, only the binary valence scale was used, where levels one to five were considered as low valence (unpleasant) and levels six to nine as high valence (pleasant). The database is available online http://www.ibug.doc.ic.ac.uk/resources/mahnob-hci-tagging-database/.

The multimodal emotion recognition (valence) pipeline starts with face detection in video, followed by face landmark detection, features extraction from face and peripheral signals, and ends with training and signals classification while using NeuCube.

### 5.2. Face Detection and Tracking

The first step for analyzing face emotion recognition in video is face detection and tracking in frames. Computer Vision (CV) Matlab Toolbox was used for this task. The output of this step is the corner coordinates for the polygon enclosing the face for each frame in the video.

The face detection that was carried out in this work included the following steps,

1. The face in the first frame was detected using the *vision.CascadeObjectDetector* object in the CV toolbox. This function uses the Viola-Jones algorithm [108] to detect people’s faces, noses, eyes, mouth, or upper body. It outputs the region of interest (ROI) for the face as a polygon, enclosing the face. Specifically, the algorithm uses the histogram-of-oriented gradients (HOG), Local Binary Patterns (LBP), Haar-like features, and a cascade of classifiers trained using boosting.
2. The corner features in the first frame ROI were detected using the *detectMinEigenFeatures* function in CV toolbox, which uses the minimum eigenvalue algorithm [109].
3. For the tracking of feature points in the remaining frames, we used the Kanade–Lucas Tomasi (KLT) algorithm [109,110].
4. Finally, in order to estimate the motion of the face, we used *estimateGeometricTransform* function in the CV toolbox to apply the same transformation to the ROI that was detected in the previous frame to obtain the ROI in the next frame.

**Figure 1:**
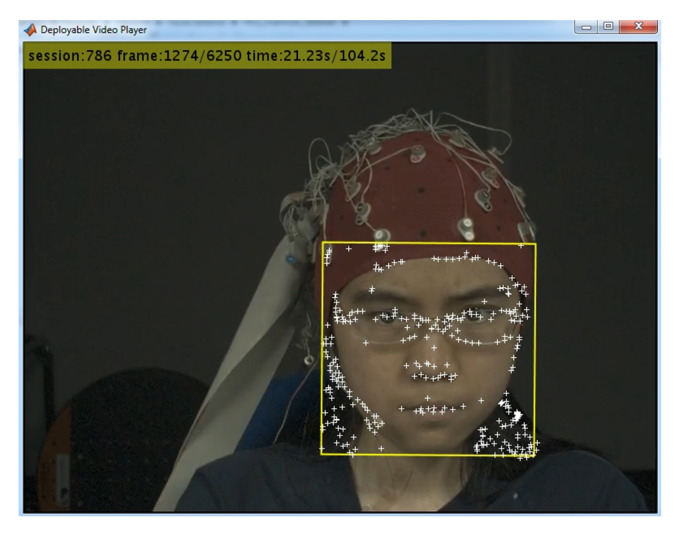
Example of face detection in Mahnob-HCI showing the feature points tracked along the video.

Figure 1 shows the output of the face detection step. We found that point tracking in frames to detect face is computationally more efficient than face detection in each frame. Furthermore, point tracking can manage problems that can emerge in face detection, such as making gestures with hand that may occlude parts of the face.

### 5.3. Face Landmarks Detection

**Figure 2:**
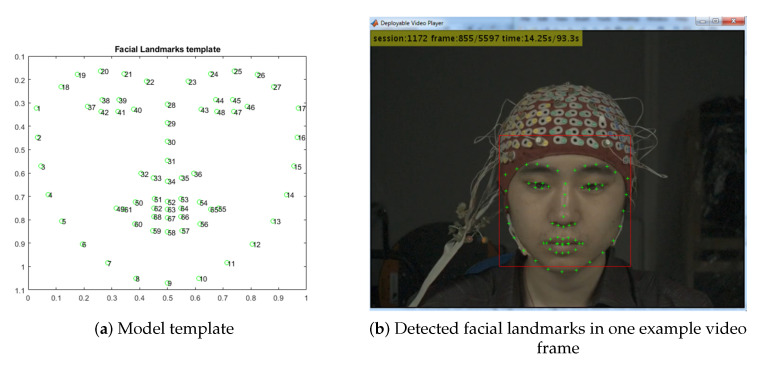
Facial landmarks detection.

Using the detected ROIs (See Section 5.2), a trained model (DLIB) for 68 facial landmarks detection was used for each frame in the video [111]. DLIB library can be obtained from http://dlib.net/files/shape_predictor_68_face_landmarks.dat.bz2. The processing time for this task was around 100 s per video (i.e., approximately 30 min per subject). Figure 2 shows the model template (a) and one example video frame with detected facial landmarks (b) adjusted to relevant facial structures (mouth, eyebrows, eyes, nose, and face borders).

### 5.4. Face Features Extraction

**Figure 3:**
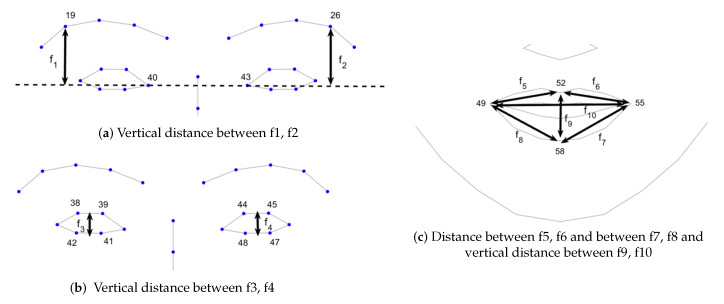
Facial features.

We extracted the following featured from facial landmarks (see Figure 3),

1. Vertical distance between the horizontal line connecting the inner corners of the eyes and outer eyebrow (f1, f2).
2. Vertical distances between the upper eyelids and the lower eyelids (f3, f4).
3. Distances between the upper lip and mouth corners (f5, f6).
4. Distances between the lower lip and mouth corners (f7, f8).
5. Vertical distance between the upper and the lower lip (f9) and distance between the mouth corners (f10)

We assume that the participants hold a neutral face for the first two seconds after starting the stimulus. Because we want to detect changes in facial features, therefore the mean features in first 2 s are subtracted from facial features for each response video.

### 5.5. Physiological Features

**Figure 4:**
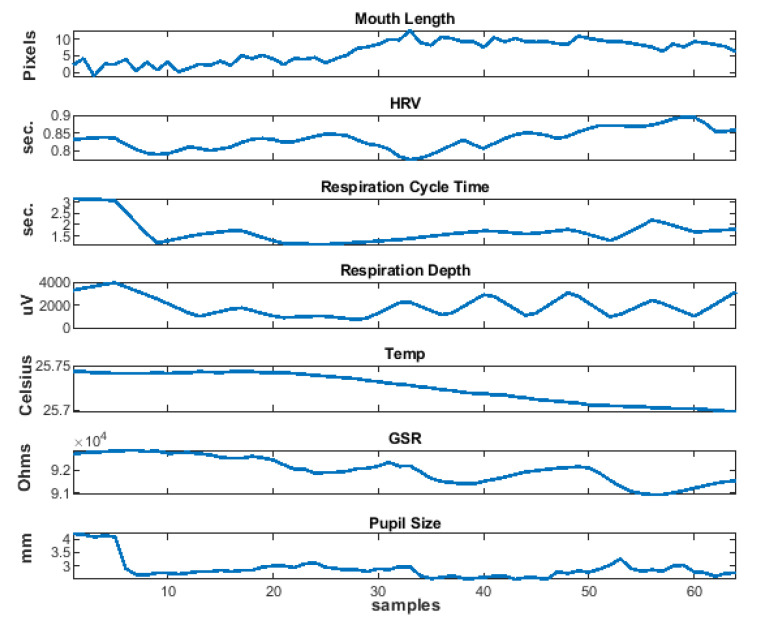
Elicited signal features in the last 30 seconds of video.

Heart rate variability (HRV), respiration variability, respiration depth, skin temperature, GSR, and pupil diameter are used as physiological features in this study. The ECG signal is pre-processed by mean subtraction and band pass filtered with a low pass and high pass filter in cascade (Least Square FIR, 70 dB, 0.05–40 Hz, 1 dB ripple) for reducing high frequency noise as muscular activation and reducing shifting due to respiration. R waves are detected using Pan and Tompkins algorithm [112] for calculating the RR interval (for HRV) as a feature. The *findpeaks.m* function in Matlab (Signal Processing Toolbox) was applied to the respiration signal to detect valleys and peaks in signal and further obtain the respiration variability (time between cycles) and respiration depth (cycle amplitude). The raw Temperature (Celsius) and GSR measurements were also considered as feature signals. Additionally, from the gaze data, the mean pupil diameter (from both eyes) was computed as an additional feature signal. Figure 4 shows examples of physiological features.

**Figure 5:**
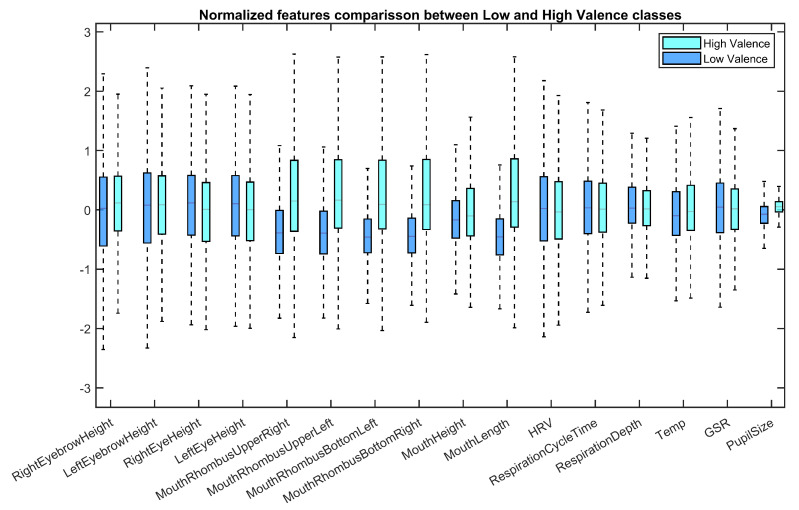
Boxplot for features in Mahnob-HCI dataset for valence emotional dimension.

All of the facial and peripheral physiological features obtained in the analyzed window (last 30 s of video) were resampled to 64 samples. All of the features are calculated in whole video response too, and resampled to 64 points, in order to capture changes in physiological feature. The first sample is subtracted from features in windows for further analysis. We suppose that this first measurement in whole video means for resting or neutral state for physiological signals. Figure 5 shows the distribution of normalized features. It can be noted that mouth-related features and pupil size have better discriminative power between low and high valence. The outliers are omitted for visualization purposes.

### 5.6. NeuCube SNN for Facial Emotion Recognition

**Figure 6:**
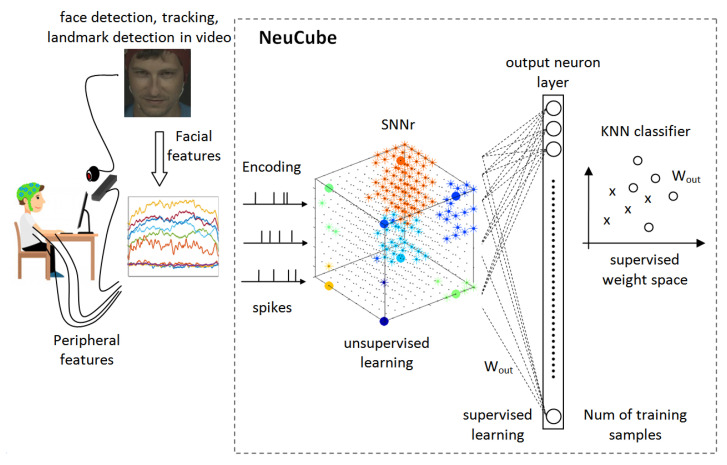
Proposed method for emotion valence classification using NeuCube.

We used NeuCube proposed in [18] to build a system for emotion valence classification. A general scheme of our approach based on NeuCube is presented in Figure 6. As described in Section 4.1, the NeuCube structure includes Encoding, 3D-SNNr, output neuron layer, and KNN classifier. Training and classifying spatio-temporal data using NeuCube have the following stages:

- **Encoding:** encode the spatio-temporal data (features) into trains of spikes.
- **SNNr:** construct a recurrent 3D SNNr and initialize the connection weights among neurons.
- **Input neurons location:** locate the input neurons in the SNNr keeping related inputs near in space.
- **Unsupervised learning:** feed the SNNr with training data to learn in an unsupervised mode the spatio-temporal patterns in the data.
- **Supervised learning:** construct an eSNN classifier to learn to classify different dynamic pattern in SNNr activities.
- **Classification:** feed the SNNr with testing data for classification purposes.

We briefly explain each stage in the following sections.

#### 5.6.1. Encoding

**Figure 7:**
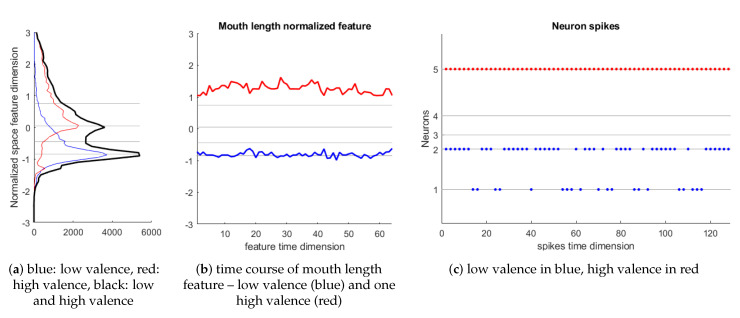
Encoding Continuous feature values to five neurons spiking.

The coding method that we used was inspired by Gaussian Receptive Field population-based sparse coding proposed in [89,90]. This method codes each continuous value from a time-based feature to spikes emitted at different times by a neuron population. The whole feature range is covered for the neurons and the time for generating the spikes depends on the distance from the current value to the center of a Gaussian receptive field covering each value interval. We used a population of five neurons per feature, in which only a neuron from the group spikes at the current time step. Figure 7 shows an example of coding the mouth length feature. Note that the dimension of feature is 64 and the temporal dimension of each spikes train is 129, because zeros are inserted between the spikes.

It can be noted from the distribution of mouth length feature (Figure 7a, left plot; blue: low valence, red: high valence, black: low and high valence), that there are two peaks in the distribution indicating the separation between the two class. In the middle plot (Figure 7b), the time course of mouth length feature in a low valence event (blue) and one high valence event (red) for the subject 1 are shown. In the right plot (Figure 7c), spikes generated for these two events are shown (low valence in blue, high valence in red). The levels that define the receptive fields or range for exciting each neuron are defined using the feature distribution in the data from all detected events for all analyzed subjects. Levels for each five neuron population are automatically obtained by analyzing the histogram in such a way that the five ranges have the same count of value occurrences. The levels are shown as gray lines (left and middle plots in Figure 7b). Note that each feature value in time produces a spike in only one neuron from the population. Eighty input neuron are allocated in NeuCube network, as we have ten facial features and six peripheral signals.

#### 5.6.2. Construction of SNNr

**Figure 8:**
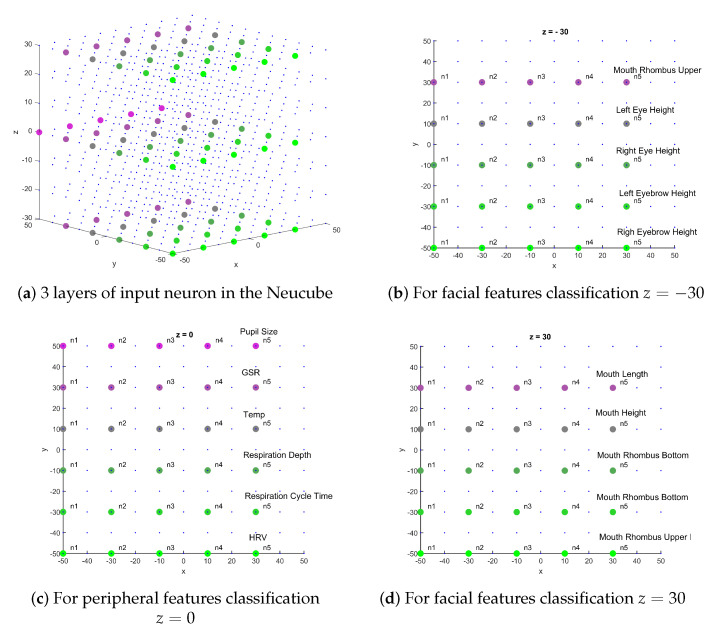
Input neurons location for facial and peripheral features classification. n1 means for the neuron coding the lowest values and n5 the highest ones for each feature. Note there are 3 layers of input neuron in the cube, located at z=−30 (facial), z=0 (peripheral), and z=30 (facial).

When brain imaging data, such as EEG, Are used, the SNNr can be built with a shape resembling the human brain [18] and the input neurons can be located based on the anatomical location of the EEG electrodes. However, in this study, as we are building a general classifier of facial features, we chose to build an 11×11×7 array of neurons (equally spaced in *x* and *y* axes), as shown in Figure 8. Each five neuron population are spatially arranged in NeuCube structure in lines, as illustrated in Figure 8; this way neighbor neurons code similar feature values favoring spatial neuron specialization.

**Figure 9:**
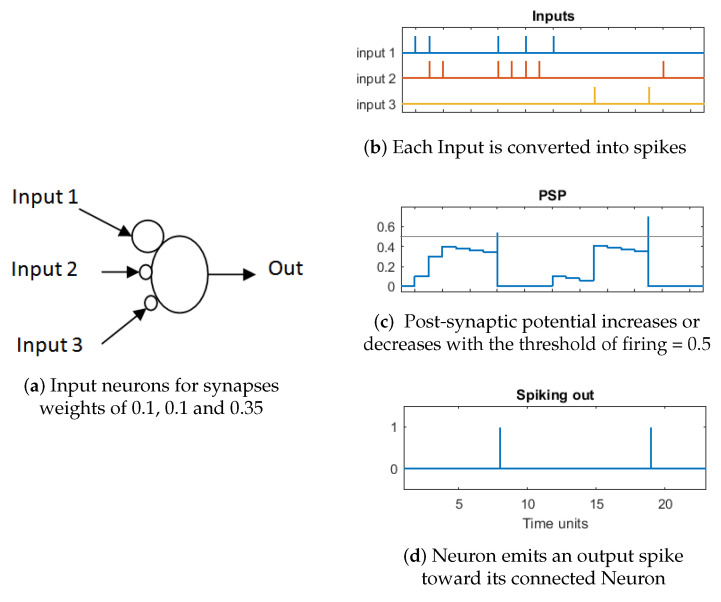
Leaky integrate-and-fire model (LIFM) neuron model. Small circles at neuron inputs represent connection weights. Note that input 1 has a bigger weight and it produces a larger effect in PSP.

The SNNr was made with leaky integrate and fire model (LIFM) spiking neurons with recurrent connections. In this neuron model, the post-synaptic potential (PSP) increases or decreases with every input spike from pre-synaptic neurons. The effect of each spike is modulated by the corresponding synaptic connection weight. If PSP reaches a specific threshold (0.5 in this work), then the neuron emits an output spike toward its connected neighbours and the PSP resets to a reference value. The PSP can leak between spikes with a predefined time constant τ, if we are using an exponential model or a constant leak time. The latter is used in this work and is set to 0.002. After a neuron spikes, the absolute refractory time (equal to 1 in this work) is simulated by disabling it to increase the PSP until a certain unit time has passed. Figure 9 shows an example of LIFM neuron simulation with a refractory time that is equal to three seconds, potential leak rate equal to 0.02, a threshold of firing that is equal to 0.5 and synapses weights of 0.1, 0.1, and 0.35. It can be noted in Figure 9 that the accumulation of spikes in time leads to an increase of PSP until a spike is generated and the effect of disregarding input spikes immediately after a spike is generated.

We set the initial connections (synapses strength) between neurons in SNNr using small-world connectivity [104,113]. The connection probability was set, such that neurons were more likely to be connected to neighboring neurons than to the distant ones. It has been shown that such an approach brings some advantages with regard to learning speed, parallel processing, and also favors the linking of specialized processing cluster units [114]. Additionally, we defined a radius *r* to be the maximum distance of connections of one neuron to another in the reservoir (r=25 in this study). The initial weights were assigned as the product of random values [−1,+1] divided by Euclidean distance between pre-synaptic and post-synaptic neuron, so that 80% of them were positive values (excitatory connection), while 20% of them were negative values (inhibitory connections). Neuron connections are unidirectional, and the direction of communication was selected randomly. Connections between input neurons and other neuron are always positive and with doubled weight in comparison with other random connections. These connections were modified in the unsupervised learning stage in order to adapt to spatio-temporal patterns in input data.

#### 5.6.3. Deep, Unsupervised SNN Training

**Figure 10:**
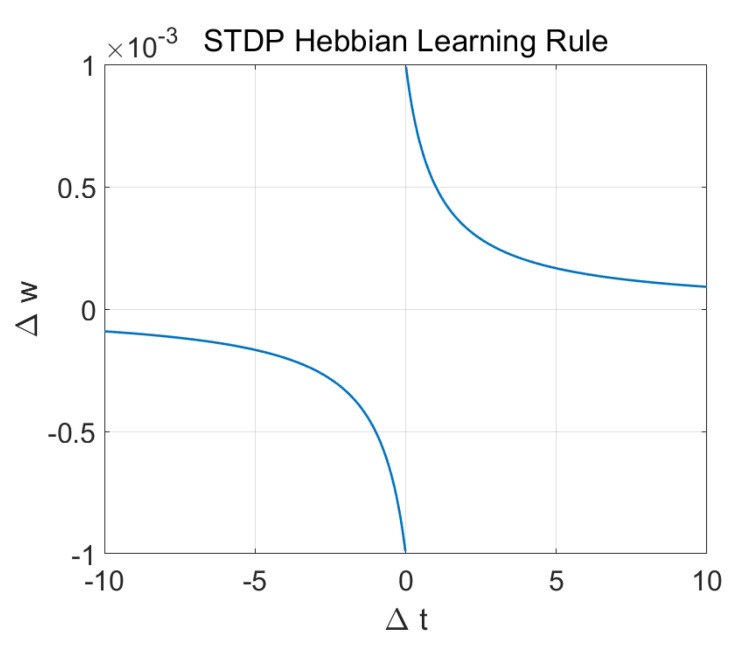
Hebbian Learning rule, connection (synaptic modification) vs difference between post- and pre-synaptic times.

We adjusted the connections between the neurons using the training data and a learning rule-based on Hebbian plasticity, called spike-time-dependent plasticity (STDP) [115]. STDP learning modifies the neuronal connection weights while taking into account the time difference between post- and pre-synaptic firing. A connection is strengthened, if postsynaptic firing occurs after presynaptic firing; otherwise, it is decreased. After STDP learning, the spatio-temporal pattern was saved in the value of connection weights in the SNNr. STDP learning rule is given as,

(1) $$\Delta w=\mathrm{sgn}\left(\Delta t\right)\frac{LR}{|\Delta t|+1}$$

where LR is the STDP Learning Rate (0.001 in this work), sgn(·) is the function sign (−1 for negative values and 1 for positive), Δt is the difference between post- and pre-synaptic times ($\Delta t=t_{\mathrm{post}}-t_{\mathrm{pre}}$) and Δw is the change in the connection weight. The Hebbian relation Δw vs Δt is depicted in Figure 10. The learning results in the creation of deep structures of connections between neurons in the SNNr.

#### 5.6.4. Supervised Output Neurons Training

The deSNN is applied for supervised learning [86]. For every single training sample, an output neuron was created and connected to all of the neurons in the trained SNNr (see Figure 6). Each output neuron was trained using the corresponding training sample by propagating the signal through the network once more. The neuron’s connections weights $w_{i,j}$ between neurons *i* (in the reservoir) and *j* (output neuron) were initially established using rank order (RO) rule [86]. The RO method ranks the order in which the first spike arrives in the *j* neuron and the weights are given as,

(2) $$w_{i,j}\left(0\right)=\alpha \;\mathrm{mod}{\;}^{\mathrm{order}(i,j)}$$

where α is a learning parameter (in a partial case, equal to 1), mod is a modulation factor that defines how important the order of the spike is (0.8 in this study), order(i,j) represents the order (the rank) of the first spike at synapse (i,j) ranked among all of the spikes arriving from all synapses to the neuron *j*. Furthermore, order(i,j)=0 for the first spike to neuron *j* and increases according to the input spike order at other synapses.

Once a synaptic weight $w_{i,j}$ is initialized, based on the order of the first spike from *i* to *j*, the synapse becomes dynamic. It increases its value with a small positive value (drift=0.005) at any time *t* a new spike arrives at this synapse and decreases its value if there is no spike at this time, as described in the following formula,

$$w_{i,j}\left(t\right)=\left\{\begin{array}{c} w_{i,j}\left(t-1\right)+drift,\;\mathrm{if}\;S_{i,j}\left(t\right)=1\\ w_{i,j}\left(t-1\right)-drift,\;\mathrm{if}\;S_{i,j}\left(t\right)=0 \end{array}\right.$$

where $S_{i,j}\left(t\right)$ describes the existence of spike from neuron *i* entering to neuron *j* at time *t*. Every generated output neuron was trained to recognize and classify spatio-temporal patterns of weights adjusted by a corresponding labeled input training sample.

#### 5.6.5. Classification

At classification stage, the NeuCube is fed with validation data. For each sample, data synaptic weights for output neurons are calculated while using the same supervised rules used in supervised training procedure. The connection weights that are learned in this process are then classified using a K-nearest neighbor (KNN, with K=3 neighbors) algorithm and the labels that are known for all of the samples.

We ran the whole NeuCube framework in a leave one subject out mode (LOSO) in order to test its capacity for learn spatio–temporal features from subjects and classify an unseen new subject.

#### 5.6.6. Fusion of Multimodal Signals

Two schemas for the fusion of multimodal signals were explored—(1) features-level and (2) decision-level fusion. For features-level fusion, we coded all of the features (facial and peripheral) and included as input in NeuCube. Regarding decision-level fusion, for each subject, we calculated the accuracy of NeuCube classification in training data (rest of subjects) for separated modalities (facial and peripheral), and we chose the method with higher accuracy as the method for doing validation classification for the specific subject.

#### 5.6.7. NeuCube Parameters

NeuCube performance in analyzing spatio-temporal data depends on several parameters. We chose a set of default parameter values that are equal to that used in the NeuCube development system publicly available online http://www.kedri.aut.ac.nz/neucube, with the exception in refractory time. We used one time unit for this parameter in order to increase neuron activity. The NeuCube parameters used in this work are given in Table 1.

## 6. Results

NeuCube framework was fed with coded data under a LOSO cross validation scheme, i.e., all of the data from a specific subject were excluded from the training set. All the parameters were fixed with values mentioned in Method section. Table 2 shows classification accuracy results in Mahnob-HCI dataset. We also included F1-score since some subject has imbalanced data, i.e., more than twice the number of sample for one class than the other. Total accuracies at the end of the table can be used for comparison with other works, because they are calculated using all the data which can be assumed as balanced, 390 videos were analyzed (207:53.07% low valence, 183:46.92% high valence).

Paired sample *t*-test when comparing the F1-score from Peripheral and Facial features result in no difference between them (p<0.05). F1-score using decision-level features does not show a difference with facial nor peripheral (p<0.05). Additionally, feature-level fusion F1-score (0.74) results in being better than decision-level fusion F1-score, 0.67 (p<0.01).

For decision-level fusion, we obtained a mean accuracy of 83.7% for classifying the training data using facial features and 80.94% using peripheral training data.

### Clustering Spike Communication

**Figure 11:**
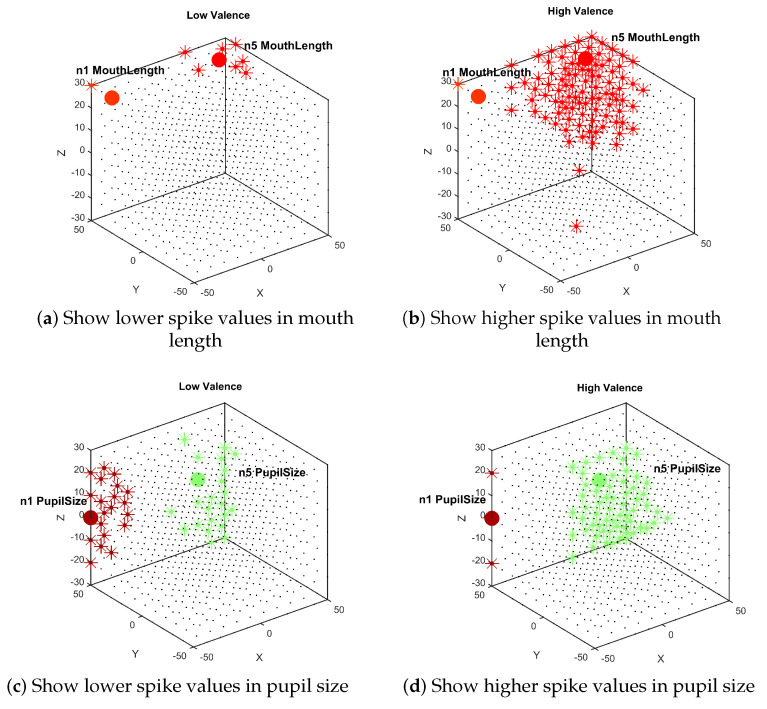
Neuron activity pattern example when NeuCube is trained using each Separate data (low and high valence).

NeuCube framework has an option to analyze clusters of neuron-surrounding input neurons using the spike amount communicated between a pair of neurons. Figure 11 shows an example using this tool when the neuron reservoir is trained separately with one class (low valence) and the other one (high valence). For visualization purposes and taking into account that mouth length and pupil size have more discriminative power regarding the rest of features, only input neurons coding higher and lower values in mouth length and pupil size are shown. Figure 11 shows that neurons coding high values of mouth length and pupil size are more active for high valence and for this reason the cluster of spiking communication surrounding these neurons are bigger. Note that neuron coding low values of pupil size is more active for low valence. These results agree with features distribution in Figure 4.

## 7. Discussion

In this work, we developed an approach based on NeuCube [18], which is an eSNN framework, in order to classify emotional valence using multimodal dataset that included video and physiological signals. We used a population coding scheme, based on ROC to encode input data into spikes, which SNNs can handle. When tested on the benchmark dataset, the MAHNOB-HCI, our approach resulted in a accuracy about 73.15% for emotion classification. To the best of our knowledge, there has not been any other study to utilize SNN for affect recognition with multimodal data. In addition to the good accuracy of classification, the SNN system can be incrementally trained on new data and new features in an adaptive way, allowing for the system to be used in an on-line applications [96].

### 7.1. Related Work

The MAHNOB-HCI dataset has been used in several studies owing to its difficulty for the classification of spontaneous emotional responses from subjects. Several studies have resorted to a multimodal approach because the MAHNOB-HCI dataset also contains multimodal data in the form of physiological and audio signals.

In a study by Koelstra and Patras [116], EEG and facial expressions were fused to perform affect recognition and implicit tagging. In case of EEG, power spectral density (PSD) features were used and for facial expression, an AU detection method was used, which was originally proposed in [117]. Basically, the AU detection was performed using Free-form Deformations (FFDs) and Motion History Images too. For facial recognition, they trained the system using the MMI dataset [118] and obtained 64.5% of binary valence classification using only facial features and 74% by combining facial and EEG features. They performed a per-subject leave-one-trial-out cross-validation, where the classifier is trained on 19 trials from the same subject and tested on the 20th. As can be seen from their study, only using facial features result in low accuracies and fusion with EEG signal improved the classification accuracy.

Boxuan and colleagues developed a temporal information preserving framework by splitting signals into multiple stages in each video. They achieved a valence (unpleasant, neutral, pleasant) classification accuracy of 54% using only facial expression and 69% when fusing with physiological signals [119]. They used Affdex SDK software [120], trained in 10,000 manually labeled facial images, which classify emotion-based on HOG features and support vector machine (SVM) classifier. Huang and colleagues obtained 50.57% for valence classification using appearance descriptors based facial features (local binary pattern from three orthogonal planes, LBP-TOP) and 66.28% using fusion it with global EEG features [121]. They used the LOSO cross-validation scheme in nine emotion categories. A convolution deep belief network (CDBN) was proposed in [122] in order to learn emotional features from multimodal datasets and the authors reported a classification accuracy of 58.5% with the MAHNOB-HCI dataset. Torres et al. [123] performed feature selection using discriminant-based algorithms, while using EEG and peripheral signals. Their results showed that EEG-related features show the highest discrimination ability. Furthermore, it was shown that EEG features, along with GSR, achieved the highest discrimination for arousal index, whereas for the valence index, EEG features are accompanied by the heart rate features in achieving the highest discrimination power. For the MAHNOB-HCI dataset, they obtain a classification accuracy of 66.09% and 69.59% in the valence and arousal dimension, respectively. Liu et al. [124] tested a deep learning approach based on multi-layer Long short-term memory recurrent neural network (LSTM-RNN) for emotion recognition, which combined temporal attention and band attention. They achieved an accuracy of 74.5% in valence classification (9 class) fusing video and EEG analysis. They used 20 participants for training, four participants for validation, and three participants for testing. Huang et al. [125] used transfer learning technique (pre-trained convolutional neural network, CNN) to obtain an 73.33% in binary valence accuracy in MAHNOB-HCI dataset using facial features and 75.21 fusing with EEG features.

Overall, as shown in Table 3, the results that we have obtained from the MAHNOB-HCI dataset are comparable with the state of the art work learning methods applied on this database. We observe that, in some cases, the classification accuracy obtained using our SNN approach is better than the ANN approach, that have also used EEG signals, which we have excluded. It is also to be noted that it is difficult to establish a fair comparison with most of the previous works, as we did not include EEG features and use pretrained models, as in [125]. We also disregarded all of the data related with the subject in a Leave-subject out validation scheme. Furthermore, in contrast with Deep Learning approaches, our SNN based method provides more interpretability of the model due to specialization of neurons clusters, needs for fewer data to train, and this can be done online with one pass of a new training sample.

### 7.2. Limitations

Our work has several limitations. First, we did not include any EEG features, because changes in EEG features that are associated with emotion are lumped features and we wanted to test NeuCube with temporal spatial patterns. The addition of raw EEG temporal signals into NeuCube would need for addition of much more input neurons into the model. We found this unfeasible to compute in a reasonable time for our experiments. Actually, temporal variations on EEG features for emotion detection are in a different scale than variation in other peripheral signals, we are using a 30 s window to analyze changes in physiological and facial features, this is a very short time to expect changes in lumped EEG features due to emotions. As discussed previously, several studies have shown that including EEG features considerably improves the classification accuracy. However, there are several challenges in using EEG for emotion recognition [126], including the selection of robust features, continous decoding of affective states, reliable decoding of long-term reliability of EEG recordings for such studies, long preparation time, and, most importantly, adopting a proper model of emotion with regard to EEG and understanding the EEG representation of affective states. For an excellent overview of these challenges the reader is directed to [126]. Nonetheless, the possibility of using EEG with the NeuCube framework will be explored in our future studies. Second, other important features that could be utilized from the multimodal data could be speech and postures. Several studies have considered the implications of including speech in affect recognition, with pitch being considered to be an index into arousal [1], although the classification accuracy is shown to be lower than facial expression. Nonetheless, this feature should definitely be considered in the future studies with SNN given the noninvasive and easy procedure to acquire voice. With regard to posture tracking, again it is a non-intrusive acquisition to the user’s experience, but the equipment requires more expensive equipment as compared to speech. Additionally, there are some constraints with regard to the user’s position, for example the user should be sitting [1].

We have also assumed that the face that is captured during the first two seconds after the stimulus is presented is neutral and consider it as the baseline. This could be problematic, especially if the participant is tired. Because we chose the last 30 s window for event selection in each video, we do not take into account the long-lasting facial expression. It could be interesting if long term facial variation inside the video could be considered as detected events. Additionally, it could be interesting to incorporate detecting facial micro-expressions in our framework but this is in general challenging due to limited availability of such data and as well as difficulties in analyzing minute changes in expression [127]. Few methods have been proposed to address the problem of detecting micro-expressions using spatio-temporal local texture descriptor [128], Gabor filter with SVM classifier [129], and LBP-TOP with nearest neighbor classifier [130], which can be incorporated to add more information for the SNN framework. Another improvement could be to normalize expressions between subjects by using pose estimation [131], or correction of a 3D model [132,133]. Further improvements could be made along the lines of detecting non-frontal head poses, identity bias, as well as illumination variation.

Although we studied the effect of varying certain NeuCube parameters, the performance of the proposed system may be affected by the choice of several other parameters. For instance, the effect of varying other NeuCube parameters such as radio, firing threshold, refractory time, and NeuCube resolution should be carefully investigated. The NeuCube framework also provides parameter optimization tool, which could be utilized instead of setting the parameters in an ad hoc manner.

## 8. Conclusions

Utilizing multimodal data to solve the problem of affect recognition with state of the art deep learning methods has gained a lot of popularity. SNNs offer an alternative to ANNs, where, in the former, is biologically more realistic model of neurons. In this work, we proposed a novel solution of using a variant of SNN, known as NeuCube, which is an eSNN, to solve affect recognition problem using multimodal data obtained from MAHANOB-HCI dataset. The eSNN is based on the ECOS principles which includes, efficient processing of spatio-temporal data and open evolving structure. Despite not including EEG, our approach provided results comparable to deep learning methods that utilize multimodal data, including EEG. In addition to the good accuracy of classification, the SNN system can be incrementally trained on new data and new features in an adaptive way, allowing for the system to be used in on-line applications.

## Figures and Tables

**Table 1:** NeuCube parameters.

Small world radius (*r*)	25
STDP learning rate (LR)	0.001
Threshold of firing	0.5
Potential leak rate	0.002
Refractory time	1 s
mod	0.84
drift	0.005
K	3

**Table 2:** Video valence classification accuracy in Mahnob-HCI dataset using NeuCube.

Subject ID	Facial Features Accuracy (%)	Physiological Features Accuracy (%)	Fusion Detection Accuracy (%)	Fusion Features (%)
1	73.33	66.67	66.67	73.33
2	62.5	50	62.5	56.25
3	75	72.73	75	81.82
4	78.57	66.67	66.67	83.33
5	75	50	75	68.75
6	58.82	70.59	58.82	70.59
7	75	60	75	93.33
8	64.29	50	64.29	64.29
9	60	100	60	90
10	61.54	69.23	61.54	69.23
11	78.57	61.54	61.54	76.92
13	64.29	71.43	64.29	85.71
14	50	57.14	50	71.43
16	72.73	63.64	63.64	63.64
17	62.5	80	62.5	40
18	41.67	50	41.67	62.5
19	61.54	75	61.54	58.33
20	53.33	73.33	53.33	80
21	66.67	71.43	66.67	71.43
22	66.67	66.67	66.67	80
23	75	50	75	75
24	69.23	53.85	69.23	76.92
25	66.67	77.78	66.67	55.56
27	68.75	60	68.75	80
28	66.67	66.67	66.67	86.67
29	68.75	50	68.75	64.29
30	78.57	57.14	78.57	78.57
Total	66.67	63.84	65.11	73.15

**Table 3:** Comparison with related works on valence classification using Mahnob-HCI dataset.

Works	Features	Method	Classes	Cross-Validation	Accuracy %
[116] Koelstra	Facial + EEG	Free-form Deformation and Motion History Images	Binary valence	Trained with MMI dataset, and data from the same subject	74
[119] Zhong	Facial + Physiological	Temporal Information Preserving Framework, SVM	Valence (3 classes)	LOSO	69
[121] Huang	Facial + EEG	LBP-TOP, Transfer learning CNN, SVM	9 emotion categories	LOSO	62.28
[122] Ranga- nathan	Facial + Body + Physiologic	Convolutional deep belief network (CDBN) and SVM	Not mentioned	LOSO	58.5
[123] Torres- Valencia	EEG + Peripheral	Discriminant-based algorithm, SVM	Binary valence	80% train data-20% test data	66.09
[124] Liu	Facial + EEG	LSTM-RNN	Valence, 9 classes	24 subject training and 3 for testing	74.5
[125] Huang	Facial + EEG	Pretrained CNN	Binary valence	LOSO	75.21
ours	Facial + Peripheral	SNN, feature-level fusion	Binary valence	LOSO	73.15
